# Exploration of Fully‐Automated Body Composition Analysis Using Routine CT‐Staging of Lung Cancer Patients for Survival Prognosis

**DOI:** 10.1002/jcsm.70021

**Published:** 2025-08-06

**Authors:** Marc‐David Künnemann, Christian Römer, Anne Helfen, Annalen Bleckmann, Marcel Kemper, Walter Heindel, Tobias J. Brix, Michael Forsting, Johannes Haubold, Marcel Opitz, Martin Schuler, Felix Nensa, Katarzyna Borys, René Hosch

**Affiliations:** ^1^ Clinic for Radiology University of Münster and University Hospital Münster Münster Germany; ^2^ West German Cancer Center (WTZ) University Hospital Münster Münster Germany; ^3^ Department of Medicine A, Hematology, Oncology, and Pneumology University Hospital Münster Münster Germany; ^4^ Institute of Medical Informatics University of Münster Münster Germany; ^5^ Institute of Diagnostic and Interventional Radiology and Neuroradiology University Hospital Essen Essen Germany; ^6^ Institute for Artificial Intelligence in Medicine (IKIM) University Hospital Essen Essen Germany; ^7^ West German Cancer Center (WTZ), Department of Medical Oncology University Hospital Essen Essen Germany; ^8^ National Center for Tumor Diseases (NCT), NCT West, a Partnership of DKFZ, University of Duisburg‐Essen and University Hospital Essen University of Köln and University Hospital Köln Essen Germany

**Keywords:** body composition analysis, deep learning, lung cancer, myosteatosis, sarcopenia

## Abstract

**Background:**

AI‐driven automated body composition analysis (BCA) may provide quantitative prognostic biomarkers derived from routine staging CTs. This two‐centre study evaluates the prognostic value of these volumetric markers for overall survival in lung cancer patients.

**Methods:**

Lung cancer cohorts from Hospital A (*n* = 3345, median age 65, 86% NSCLC, 40% M1, 40% female) and B (*n* = 1364, median age 66, 87% NSCLC, 37% M1, 38% female) underwent automated BCA of abdominal CTs ±60 days of primary diagnosis. A deep learning network segmented muscle, bone and adipose tissues (visceral = VAT, subcutaneous = SAT, intra‐/intermuscular = IMAT and total = TAT) to derive three markers: Sarcopenia Index (SI = Muscle/Bone), Myosteatotic Fat Index (MFI = IMAT/TAT) and Abdominal Fat Index (AFI = VAT/SAT). Kaplan–Meier survival analysis, Cox proportional hazards modelling and machine learning‐based survival prediction were performed. A survival model including clinical data (BMI, ECOG, L3‐SMI, ‐SATI, ‐VATI and ‐IMATI) was fitted on Hospital A data and validated on Hospital B data.

**Results:**

In nonmetastatic NSCLC, high SI predicted longer survival across centres for males (Hospital A: 24.6 vs. 46.0 months; Hospital B: 13.3 vs. 28.9 months; both *p* < 0.001) and females (Hospital A: 37.9 vs. 53.6 months, *p* = 0.008; Hospital B: 23.0 vs. 28.6 months, *p* = 0.018). High MFI indicated reduced survival in males at both hospitals (Hospital A: 43.7 vs. 28.2 months; Hospital B: 28.8 vs. 14.3 months; both *p* ≤ 0.001) but showed center‐dependent effects in females (significant only in Hospital A, *p* < 0.01). In metastatic disease, SI remained prognostic for males at both centres (*p* < 0.05), while MFI was significant only in Hospital A (*p* ≤ 0.001) and AFI only in Hospital B (*p* = 0.042). Multivariate Cox regression confirmed that higher SI was protective (A: HR 0.53, B: 0.59, *p* ≤ 0.001), while MFI was associated with shorter survival (A: HR 1.31, B: 1.12, *p* < 0.01).

The multivariate survival model trained on Hospital A's data demonstrated prognostic differentiation of groups in internal (*n* = 209, *p* ≤ 0.001) and external (Hospital B, *n* = 361, *p* = 0.044) validation, with SI feature importance (0.037) ranking below ECOG (0.082) and M‐status (0.078), outperforming all other features including conventional L3‐single‐slice measurements.

**Conclusion:**

CT‐based volumetric BCA provides prognostic biomarkers in lung cancer with varying significance by sex, disease stage and centre. SI was the strongest prognostic marker, outperforming conventional L3‐based measurements, while fat‐related markers showed varying associations. Our multivariate model suggests that BCA markers, particularly SI, may enhance risk stratification in lung cancer, pending centre‐specific and sex‐specific validation. Integration of these markers into clinical workflows could enable personalized care and targeted interventions for high‐risk patients.

**Abbreviations**
BCA: body composition analysis
CT: computed tomography
IMAT: intra‐and intermuscular adipose tissue
NSCLC: non‐small cell lung cancer
SAT: subcutaneous adipose tissue
SCLC: small cell lung cancer
TAT: total adipose tissue
VAT: visceral adipose tissue
WTZ: West German Tumor Center

## 1 Introduction

Lung cancer, including small cell lung cancer (SCLC) and non‐small cell lung cancer (NSCLC), is a prevalent cancer type with the highest cancer‐related mortality rate worldwide [1], posing a significant healthcare burden attributed to late‐stage diagnosis and the intricacies of effective treatment [2].

Navigating treatment complexities and personalizing care remain challenging but hold significant potential to improve prognosis [3, 4]. For example, differences in body composition among cancer patients may contribute to variations in the metabolism of cytotoxic agents [5]. We aim to identify reliable prognostic biomarkers that can support sex‐specific patient stratification [6] and guide clinical decision‐making, potentially reducing mortality and improving quality of life. While we do not specifically address cancer progression or therapy outcome predictions in this study, we estimate our findings provide a foundation for future investigations exploring interactions between body composition, therapeutic interventions and disease outcomes.

Amidst these challenges and opportunities, computed tomography (CT) body composition analysis (BCA) shows promise by providing detailed patient‐specific morphological information [7] without additional radiation exposure or procedural burden, as it is already part of routine tumour staging. Several studies have explored this approach, primarily focusing on single‐slice analysis at the L3 vertebral level.

In a comprehensive review, Abbass et al. [8] analysed 20 CT studies with 10807 patients, finding consistent associations between two BCA markers (skeletal muscle index and skeletal muscle density) at the L3 level and systemic inflammatory response across cancer types. Nakamura et al. [9] associate sarcopenia at the L3 level with poorer 5‐year survival rates and postoperative complications in NSCLC patients. Similarly, Kazemi‐Bajestani et al. [10] establish a connection between cancer cachexia measured at the L3 level and adverse outcomes in various cancers. Despite these advancements, Troschel et al. [11] highlight the need for standardized markers, especially for sarcopenia, to refine prognostication and treatment assignment.

The focus of our research is to apply and validate fully‐automated volumetric CT BCA to develop prognostic biomarkers in lung cancer.

Through outcome‐based correlation analyses, we evaluate the prognostic and sex‐specific value of multiple abdominal body composition biomarkers in patients in both NSCLC and SCLC across two institutions. While we maintained centre separation for independent biomarker validation in our primary analyses, we also evaluated a predictive model developed using Hospital A's data, tested using Hospital B's cohort, to assess its real‐world prognostic performance across institutionally heterogeneous populations.

To the best of our knowledge, no work has yet been established for the survival analysis of NSCLC or SCLC patients with respect to multiple biomarkers derived from automated volumetric BCA of the abdominal cavity.

## 2 Methods

### 2.1 Dataset

We conducted a comprehensive review of the oncological registry at the West German Cancer Centers Essen and Münster, isolating cases of SCLC and NSCLC diagnosed between January 2000 and November 2022. The study series selection followed a two‐step filtration process, initially based on study descriptions pertaining to abdominal and whole body CT scans, and then refined to include series covering the entire abdominal cavity, with specific requisites as follows: maximum slice thickness ≤ 5 mm, soft tissue kernel requirement, axial views only, no maximum intensity projections or scout scans and study dates within a 60‐day window around the primary diagnosis date. The data selection was agnostic of CT vendor, model and reconstruction algorithm. The study closest to the primary diagnosis date was chosen for patients with multiple applicable studies. Both native and contrasted images were considered. In addition, ECOG performance status, patient height and weight were retrieved where available, with ECOG and weight considered eligible if measured within a 60‐day window around the CT date.

### 2.2 BCA

**FIGURE 1:**
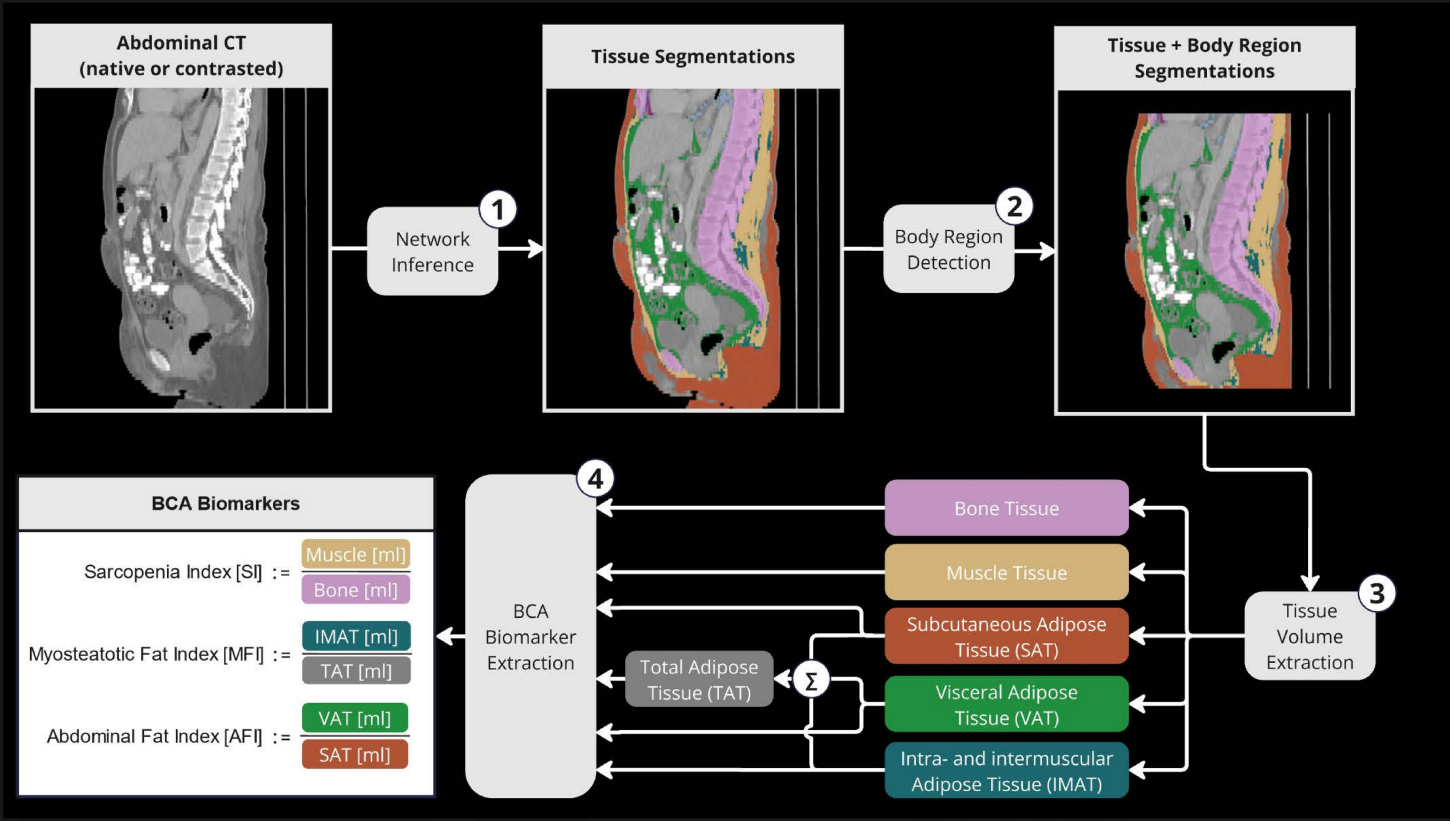
Overview of the body composition analysis (BCA) inference and marker extraction. Routine CT abdominal or whole‐body scans were introduced as input to the network. Muscle, bone and multiple adipose tissues were extracted from a uniform body region (abdominal cavity) in millilitres. In a final step, the extracted volumes were combined into specific markers: Sarcopenia Index (SI), Myosteatotic Fat Index (MFI) and Abdominal Fat Index (AFI).

The acquisition of body composition parameters was conducted using the network of Haubold et al., which allows for the extraction and quantification of bone, muscle and different adipose tissues including subcutaneous adipose tissue (SAT), visceral adipose tissue (VAT), intra‐ and intermuscular adipose tissue (IMAT) and specific body regions (thoracic cavity, abdominal cavity, etc.). The method reports statistics regarding radiation attenuation (in HU) and volume (mL) per tissue and body region. We used the tissue volume exclusively to mitigate differences between CT vendors and protocols. For this study, the abdominal cavity was defined as a uniform body region for BCA feature extraction, as presented in Figure 1.

BCA markers were extracted from the segmented tissues. For Hospital B, the BCA algorithm was packaged into a module and executed using KAAPANA/JIP [12].

### 2.3 Selection of Body Composition Markers

Body composition plays a crucial role in cancer prognosis, influencing treatment response and overall survival. Detecting early changes in body composition is essential for optimizing treatment strategies [13]. Traditional metrics, such as Body Mass Index (BMI), do not capture nuanced differences in muscle and fat distribution, which are increasingly recognized as prognostic factors in oncologic outcomes of lung cancer, especially in NSCLC [14, 15] [S6]. Therefore, we selected three quantitative imaging markers based on their established relevance in cancer‐related metabolic changes.

The Sarcopenia Index (SI) serves as a surrogate for sarcopenia, a condition characterized by progressive loss of skeletal muscle mass and function, which is associated with reduced survival, increased treatment toxicity and functional decline in cancer patients [16, 17]. Sarcopenia can occur independently or as part of cachexia, a multifactorial syndrome involving muscle and fat loss due to systemic inflammation or metabolic dysregulation [18].

In this study, SI was defined as the ratio of muscle volume (mL) to bone volume (mL):

$$\text{Sarcopenia Index}\;\left(\mathrm{SI}\right)=\frac{\text{Muscle}\;\left[\mathrm{mL}\right]}{\text{Bone}\;\left[\mathrm{mL}\right]}$$

Another common phenomenon in cancer patients is myosteatosis, also known as muscle fat infiltration, which is correlated with insulin resistance [19] [S9] and impaired survival [20] [S10]. The Myosteatotic Fat Index (MFI) indicates the relative amount of muscle fat infiltrating the muscle and was calculated as the percental proportion of IMAT volume (mL) relative to TAT volume (mL):

$$\begin{array}{c} \begin{array}{c} \text{Myosteatotic}\;\mathrm{Fat}\;\text{Index}\;\left(\mathrm{MFI}\right)=\frac{\text{Intra}-\text{and}\;\text{intermuscular}\text{ A}\text{dipose}\text{ T}\text{issue}\;\left[\mathrm{mL}\right]}{\text{Total}\;\text{Adipose}\;\text{Tissue}\;\left[\mathrm{mL}\right]}*100 \end{array} \end{array}$$

Fat distribution plays a significant role in metabolic regulation and inflammation, with sex‐specific implications for lung cancer survival [21]. SAT is more prevalent in women, exhibits lower metabolic activity and is associated with fewer health risks. In contrast, VAT, which is more prevalent in men and postmenopausal women, is metabolically active and associated with insulin resistance, cardiovascular disease and systemic inflammation [22, 23]. To quantify this balance, we introduced the Abdominal Fat Index (AFI), which represents the ratio of VAT to SAT volume (mL):

$$\text{Abdominal}\;\mathrm{Fat}\;\text{Index}\;\left(\mathrm{AFI}\right)=\frac{\text{Visceral Adipose Tissue}\;\left[\mathrm{mL}\right]}{\text{Subcutaneous Adipose Tissue}\;\left[\mathrm{mL}\right]}$$

### 2.4 Statistical Analysis

Prior to comparative analyses between the two centres, normality testing was performed using the Shapiro–Wilk test. For the NSCLC cohort, age at scan, survival time after scan and the three markers (SI, MFI and AFI) exhibited significant deviations from normal distribution across both centres, sexes and M‐status: age (W = 0.98–1.00, *p* < 0.001), survival time in months (W = 0.56–0.83, *p* < 0.001), SI (W = 0.98–0.99, *p* < 0.001), MFI (W = 0.87–0.94, *p* < 0.001) and AFI (W = 0.89–0.96, *p* < 0.001). Given these violations of normality assumptions, data are presented as median with interquartile range (IQR), and Mann–Whitney *U* tests were employed for between‐centre comparisons of age and marker distributions. To quantify the magnitude of differences between centres, Cliff's Delta effect sizes were calculated due to the non‐normal distribution of our data. Cliff's Delta values range from −1 to +1, with absolute values of 0.147, 0.33 and 0.474 considered as small, medium and large effect sizes, respectively [24]. Survival distributions were analysed using the Kaplan–Meier method to examine differences between centres, stratified by sex and metastatic (M) status. To further evaluate the impact of prognostic variables, we dichotomized each continuous predictor at its median value within each centre‐sex‐M‐status subgroup. Patients were classified as having ‘High’ biomarker levels when their values were greater than or equal to the subgroup‐specific median, and as having ‘Low’ levels when values fell below the median. Statistical comparisons between survival curves were performed using the log‐rank test, with *p* < 0.05 considered statistically significant. This approach allowed for systematic assessment of survival patterns while accounting for key clinical and demographic factors across treatment centres. Additionally, Cox proportional hazards regression models were used to assess the impact of multiple variables on survival outcomes, providing *p*‐values, hazard ratios (HRs) and 95% confidence intervals to offer a more comprehensive understanding of the underlying relationships. In all analyses, the obtained body composition features (SI, MFI and AFI) were analysed as continuous variables.

### 2.5 Machine Learning

To validate the prognostic value of the BCA markers, a survival gradient boosting model was trained and evaluated across two independent cohorts. The model was developed using time‐to‐event data, with overall survival in months as the target outcome and death as the event indicator.

To enable a comparison of volumetric BCA markers in the presence of established slice‐based or conventional body composition metrics and clinical variables, for the machine learning approach, the dataset was restricted to patients for whom all slice‐based measurements could be retrieved. These included BMI, slice‐based markers measured at the median slice of the L3 vertebrae level (Skeletal Muscle Index [SMI], SAT Index [SATI], VAT Index [VATI], IMAT Index [IMATI]) and clinical features (metastatic status, sex, age at CT and ECOG status) [25, 26, 27, 28].

Initially, an ensemble model was trained on data from Hospital A using a fivefold cross‐validation. All BCA markers and age were incorporated as continuous variables, while the clinical features, including cancer entity (NSCLC vs. SCLC), M‐status, ECOG performance status and sex, were treated as dichotomous values. The model was designed to output a continuous risk score for each patient, representing the estimated hazard. Consequently, during internal and external testing, each patient received five risk score predictions (one from each fold), which were subsequently averaged to form a single risk score per individual.

These scores were further incorporated into Kaplan–Meier survival analyses to examine their association with overall survival across both cohorts. To quantify the prognostic value of the derived risk score, we performed univariate Cox proportional hazards regression using the prediction score as a continuous variable in both internal and external validation datasets. Additionally, permutation‐based feature importance analysis was performed to identify the most influential variables contributing to the risk estimates.

All statistical analysis and machine learning methods were performed using Python 3.10.8, pandas 1.5.3, scipy 1.10.1, numpy 1.24.3, scikit‐learn 1.2.2 and lifelines 0.27.4.

## 3 Results

### 3.1 Patient Characteristics

This retrospective cohort study encompasses data from 4709 patients diagnosed with lung cancer across two hospitals, delineating 4071 cases of NSCLC and 638 cases of SCLC. The sample is stratified by sex, with a majority of males (2830 males vs. 1879 females). Subsequent analyses focus exclusively on the NSCLC cohort due to its substantially larger sample size compared to SCLC, providing greater statistical power. Additionally, the typically aggressive clinical course and abbreviated survival trajectory of SCLC limit the ability to meaningfully differentiate prognostic factors that may be more readily detected in the relatively extended survival patterns observed in NSCLC patients. Results for the SCLC cohort are available in the Supporting Information, but are not being referenced further.

At Hospital A, the NSCLC cohort comprises 2873 individuals, with a male majority (1721 males vs. 1152 females), with a slightly higher median age for men (67 [60–73] years) than for women (64 [58–71]). The metastatic status at diagnosis (M0: nonmetastatic or M1: metastatic) reveals more nonmetastatic cases (1794 vs. 1079). The SI, for which low levels indicate muscle depletion, was higher in males (median 2.26 IQR [2.00–2.53]) compared to females (median 2.10 IQR [1.89–2.31]). Similarly, the MFI, which indicates muscle adiposis, was elevated in males (2.44 [2.04–2.90]) versus females (2.22 [1.85–2.71]). The AFI, the ratio between subcutaneous and VAT, showed a similar pattern (male: 0.79 [0.62–1.00], female: 0.35 [0.27–0.45]). Hospital B's cohort for NSCLC, totalling 1198 patients, shows a similar sex distribution (747 m/451 f) and median age at diagnosis (66 [60–74] m/64 [57–71] f). Mann–Whitney *U* testing revealed no statistically significant differences in age distribution between centres when stratified by sex (*p* = 0.81 for men, *p* = 0.73 for women). The marker distributions for Hospital B are not statistically equal to Hospital A for the MFI (both sex) (2.60 [2.11–3.10] m/2.37 [1.94–3.01] f, Mann–Whitney *U* test *p* < 0.001) and SI in females (2.15 [1.90–2.41], *p* < 0.01). Despite statistical significance, the effect sizes were small (Cliff's δ = MFI 0.11 m/0.13 f, SI 0.07 f), suggesting that these differences are unlikely to be clinically meaningful. Other marker distributions are statistically equal to Hospital A (Hospital B SI: 2.23 [1.97–2.51] *p* = 0.47 m, AFI: 0.79 [0.63–1.00] *p* = 0.55 m/0.34 [0.27–0.43] *p* = 0.20 f).

Survival outcomes were compared a priori between Hospitals A and B, stratified by sex and metastatic status (M0/M1) in NSCLC patients. Kaplan–Meier analysis revealed generally shorter median survival times in Hospital B compared to Hospital A. In male patients with nonmetastatic disease (M0), median survival was significantly lower in Hospital B (19.5 months, 95% CI: 15.9–24.4) than in Hospital A (32.9 months, 95% CI: 29.0–38.3; *p* ≤ 0.001). Similarly, male patients with metastatic disease (M1) showed significantly reduced survival in Hospital B (8.5 months, 95% CI: 7.4–10.3) versus Hospital A (11.8 months, 95% CI: 9.9–13.1; *p* = 0.001). Among female M0 patients, Hospital B demonstrated significantly shorter median survival (25.9 months, 95% CI: 19.7–30.8) compared to Hospital A (48.3 months, 95% CI: 40.0–57.9; *p* ≤ 0.001). For female M1 patients, although median survival was lower in Hospital B (13.8 months, 95% CI: 10.7–17.5) than in Hospital A (17.9 months, 95% CI: 13.6–22.2), this difference did not reach statistical significance (*p* = 0.086).

Further age‐related patient characteristics are presented in Table 1.

**TABLE 1:** Descriptive patient characteristics of both hospitals' NSCLC cohorts separated by sex and accumulated (all) and M‐status. The total number of patients is indicated as total, and the distribution of the BCA markers is displayed as median and IQR.

Patient characteristics		Hospital A			Hospital B		
		Male	Female	All	Male	Female	All
M0	Patients [n]	1079	715	1794	485	281	766
	Age at diagnosis (IQR)	67.0 [61.0–73.0]	65.0 [58.0–72.0]	66.0 [60.0–73.0]	68.0 [61.0–74.0]	65.0 [59.0–71.0]	67.0 [60.0–74.0]
	Sarcopenia Index (IQR)	2.24 [2.00–2.52]	2.10 [1.87–2.30]	2.18 [1.94–2.44]	2.20 [1.95–2.49]	2.14 [1.85–2.38]	2.18 [1.92–2.45]
	Myosteatotic Fat Index (IQR)	2.47 [2.06–2.91]	2.24 [1.87–2.74]	2.39 [1.98–2.85]	2.67 [2.17–3.15]	2.36 [1.94–3.06]	2.60 [2.08–3.13]
	Abdominal Fat Index (IQR)	0.79 [0.63–1.01]	0.36 [0.28–0.45]	0.60 [0.39–0.88]	0.81 [0.64–1.01]	0.34 [0.27–0.44]	0.63 [0.39–0.89]
	ECOG	0 [0–1]	0 [0–1]	0 [0–1]	1 [0–2]	1 [0–1]	1 [0–2]
	Median survival time (months)	32.9 [29.0–38.3]	48.3 [40.0–57.9]	38.0 [33.3–42.6]	19.5 [15.9–24.4]	25.9 [19.7–30.8]	21.7 [18.9–25.3]
	1‐year survival rate	77.4%	82.5%	79.3%	61.3%	69.6%	64.2%
	5‐year survival rate	32.4%	44.2%	36.8%	23.5%	25.2%	24.1%
M1	Patients [n]	642	437	1079	262	170	432
	Age at diagnosis	65.0 [58.0–72.0]	63.0 [57.0–69.0]	64.0 [58.0–71.0]	64.0 [58.2–72.0]	62.0 [54.0–69.0]	64.0 [57.0–70.0]
	Sarcopenia Index (IQR)	2.28 [2.01–2.57]	2.11 [1.91–2.35]	2.19 [1.97–2.46]	2.29 [2.07–2.60]	2.15 [1.95–2.47]	2.23 [2.01–2.53]
	Myosteatotic Fat Index (IQR)	2.37 [2.00–2.87]	2.20 [1.84–2.63]	2.29 [1.93–2.80]	2.43 [1.98–2.93]	2.38 [1.90–2.91]	2.41 [1.96–2.92]
	Abdominal Fat Index (IQR)	0.79 [0.60–0.96]	0.34 [0.27–0.45]	0.57 [0.37–0.84]	0.77 [0.61–0.98]	0.34 [0.26–0.42]	0.59 [0.36–0.84]
	ECOG	1 [0–1]	1 [0–1]	1 [0–1]	1 [0–2]	1 [0–2]	1 [0–2]
	Median survival time (months)	11.8 [9.9–13.1]	17.9 [13.6–22.2]	13.2 [11.9–15.5]	8.5 [7.4–10.3]	13.8 [10.2–17.5]	10.3 [8.9–11.5]
	1‐year survival rate	48.8%	59.2%	53.0%	36.5%	54.3%	43.5%
	5‐year survival rate	14.0%	19.1%	16.2%	8.3%	13.7%	10.4%

### 3.2 Univariate Analysis

**FIGURE 2:**
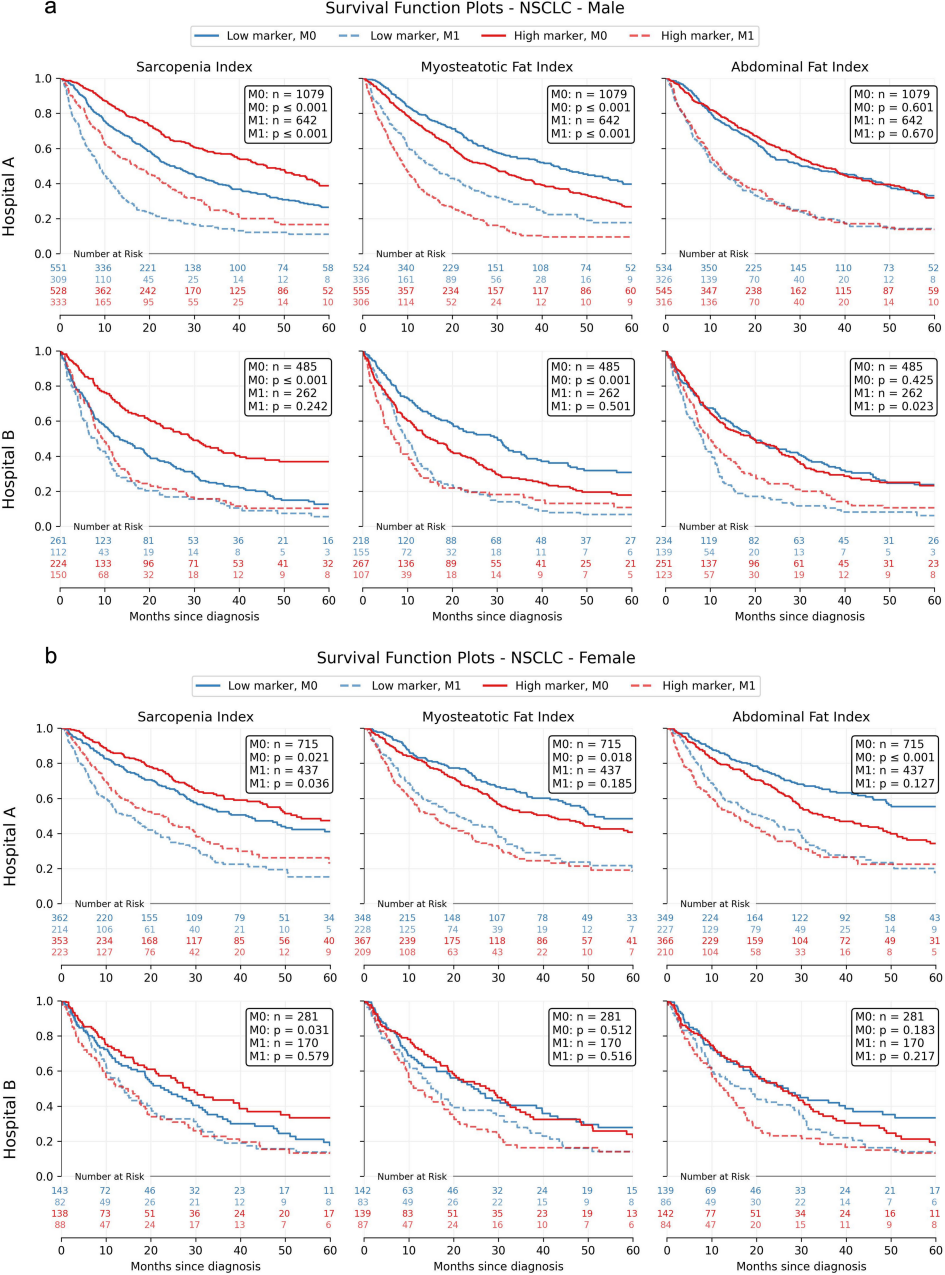
Kaplan–Meier survival curves for NSCLC patients by Sarcopenia Index, Myosteatotic Fat Index and Abdominal Fat Index at Hospitals A and B, stratified by sex and M‐status. The curves demonstrate survival probabilities over time. The significance of differences between groups with markers below and above the median is indicated by the log‐rank test *p*‐values. NSCLC: non‐small cell lung cancer.

In the univariate setting, Kaplan–Meier survival analyses were performed to examine the impact of SI, MFI and AFI on the survival of patients with NSCLC and SCLC across both hospitals, stratified by sex and M‐status. The results of the Kaplan–Meier analysis for the NSCLC cohort are presented in Figure 2.

In nonmetastatic (M0) male patients, both SI and MFI were significantly associated with survival regardless of treatment centre. Patients with high SI levels demonstrated longer median survival (24.6 vs. 46.0 months for low and high marker groups, respectively, *p* ≤ 0.001) in Hospital A and (13.3 vs. 28.9 months, *p* ≤ 0.001) in Hospital B compared to those with low SI. Conversely, high MFI levels correlated with reduced survival in both Hospital A (43.7 vs. 28.2 months, *p* ≤ 0.001) and Hospital B (28.8 vs. 14.3 months, *p* ≤ 0.001). AFI showed no significant association with survival in male M0 patients at either centre (Hospital A: *p* = 0.628; Hospital B: *p* = 0.329).

Among nonmetastatic (M0) female patients, SI was a significant predictor of survival in both treatment centres, with high levels associated with improved outcomes (Hospital A: 37.9 vs. 53.6 months, *p* = 0.008; Hospital B: 23.0 vs. 28.6 months, *p* = 0.018). MFI and AFI demonstrated centre‐specific effects, with significantly lower survival for high levels only observed in Hospital A (MFI: 53.6 vs. 38.5 months, *p* = 0.009; AFI: 69.3 vs. 33.7 months, *p* ≤ 0.001) but not in Hospital B (MFI: *p* = 0.450; AFI: *p* = 0.238).

In metastatic (M1) male patients, the association between biomarkers and survival varied by treatment centre. SI was a significant predictor in both Hospitals A (8.9 vs. 16.8 months, *p* ≤ 0.001) and B (7.0 vs. 10.4 months, *p* = 0.037). MFI was only significant in Hospital A (15.8 vs. 9.0 months, *p* ≤ 0.001), but not in B (*p* = 0.356), while AFI was significant only in Hospital B (8.1 vs. 9.6 months, *p* = 0.042) but not in A (*p* = 0.703).

For metastatic (M1) female patients, only SI in Hospital A demonstrated a significant association with survival (15.8 vs. 22.8 months, *p* = 0.032). Neither MFI nor AFI showed significant survival differences in either treatment centre for this patient subgroup (Hospital A: MFI *p* = 0.142, AFI *p* = 0.263; Hospital B: SI *p* = 0.650, MFI *p* = 0.676, AFI *p* = 0.187).

### 3.3 Multivariate Analysis

In addition to the univariate analysis, an adjusted Cox regression was conducted using age (at date of CT examination), sex and M‐status for each marker. The results of the Cox regressions performed for NSCLC at both hospitals are presented in Table 2.

**TABLE 2:** Results of the multivariate Cox regression adjusted for each BCA marker using the clinical features age at CT, sex and M‐status. For both cancer entities, the results of both sites are compared using the respective hazard ratio, 95% confidence intervals and the *p*‐value. SI, MFI and AFI were analysed as continuous variables.

Cancer entity	Hospital	Marker	Hazard ratio (95% CI)	*p*‐value	*n*
NSCLC	A	SI	0.53 (0.46–0.62)	≤ 0.001	2873
		MFI	1.31 (1.22–1.40)	≤ 0.001	
		AFI	1.01 (0.81–1.26)	0.929	
	B	SI	0.59 (0.48–0.72)	≤ 0.001	1198
		MFI	1.12 (1.04–1.21)	0.002	
		AFI	0.90 (0.70–1.16)	0.419	

High SI values demonstrated a protective effect, with HRs below 1 in both Hospital A (HR = 0.53, 95% CI = 0.46–0.62, *p* ≤ 0.001) and Hospital B (HR = 0.59, 95% CI = 0.48–0.72, *p* ≤ 0.001).

Conversely, high MFI was associated with worse survival outcomes, with HRs exceeding 1 in both Hospital A (HR = 1.31, 95% CI = 1.22–1.40, *p* ≤ 0.001) and Hospital B (HR = 1.12, 95% CI = 1.04–1.21, *p* = 0.002).

AFI did not demonstrate statistical significance as a prognostic factor in either Hospital A (HR = 1.01, 95% CI = 0.81–1.26, *p* = 0.929) or Hospital B (HR = 0.90, 95% CI = 0.70–1.16, *p* = 0.419) after adjustment for clinical covariates.

### 3.4 Machine Learning

**FIGURE 3:**
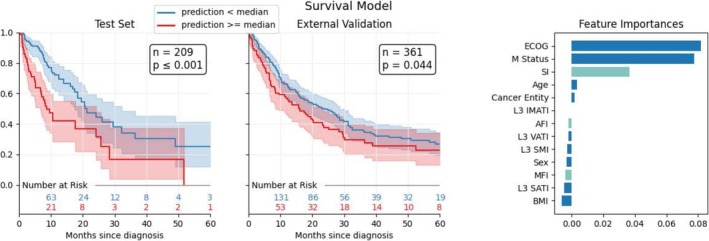
Kaplan–Meier survival curves and feature importance for survival models. (A) Test set evaluation for the survival model trained on the Hospital A data with a total of 209 patients, showing significant survival differences (*p* ≤ 0.001) between groups divided by the predicted risk score median. (B) External validation of the survival model at Hospital B with 361 patients, demonstrating consistent survival differences (*p* = 0.044). (C) Feature importance for the survival model trained on Hospital A data.

The results of the survival model trained using data from Hospital A (*n* = 485) and tested on data from Hospital A (*n* = 209) and external data from Hospital B (*n* = 361) indicate significant prognostic differentiation between patient groups divided by the median risk score for both test sets at Hospitals A and B, as shown in Figure 3.

In Hospital A's test set, survival probabilities diverge substantially between groups below and above the median, with *p* ≤ 0.001 indicating statistical significance. This pattern is replicated in Hospital B's external test set, where the survival curves also exhibit significant separation (*p* = 0.044) between the compared groups.

Univariate Cox regression confirmed the prediction score's prognostic value with significant hazard ratios in both Hospital A (HR = 4.21, 95% CI: 3.02–5.88, *p* < 0.001) and Hospital B (HR = 1.62, 95% CI: 1.23–2.15, *p* < 0.001), demonstrating robust performance across different patient populations.

Permutation‐based feature importance analysis based on mean decrease in accuracy identified ECOG performance status and M‐status as the strongest predictors of survival (importance scores 0.082 and 0.078, respectively). SI ranked third in importance (0.037), demonstrating substantial prognostic value among body composition markers. Patient age and cancer subtype contributed minimally (0.004 and 0.002, respectively), while the remaining features showed negative importance scores, suggesting they added no independent predictive value beyond the primary factors.

## 4 Discussion

In this retrospective cohort of 3345 internal and 1364 external lung cancer patients from two academic centres, we identified significant associations between CT‐derived body composition markers and overall survival. Kaplan–Meier analyses in particular revealed consistent survival differences in NSCLC patients based on body composition characteristics.

In nonmetastatic NSCLC patients, lower SI was associated with worse survival, while higher SI indicated a protective effect. These results align with the well‐established association between sarcopenia and poorer outcomes in cancer patients in general [29].

Interestingly, in patients with distant metastases, this association was only observed in Hospital A, suggesting potential centre‐specific or cohort size‐dependent influences. One likely explanation is that, in advanced tumour stages, other factors such as tumour burden, systemic inflammation or therapy resistance become dominant determinants of prognosis, reducing the relative impact of muscle mass on survival. However, these findings prompt further investigations in which these potential factors are taken into consideration.

Higher MFI was generally associated with poorer survival outcomes. This finding is also in line with several studies that have demonstrated a correlation between myosteatosis and worse prognostic outcomes in cancer patients [20]. No prognostically significant association could be established for female patients with distant metastasis in both centres, for any female cohort at Hospital B or for male patients with distant metastasis at Hospital B. We attribute these limitations in the female subgroups to the lower baseline muscle mass, which may reduce the measurable impact of myosteatosis, though this relationship requires further investigation. Other studies, particularly in animal models of cancer, suggest that intersex differences in muscle preservation may explain the less pronounced impact of myosteatosis in female patients observed in our NSCLC cohort [30]. Future longitudinal studies are required to determine whether these sex‐specific differences in MFI can be consistently observed. Additionally, in patients with advanced disease, the impact of muscle quality on survival may be less pronounced, as the poor overall prognosis likely outweighs its effect.

The AFI showed prognostic relevance only in specific subgroups: An AFI below the median exhibited a protective effect only in male NSCLC patients with metastasis at Hospital B and in nonmetastatic female NSCLC patients at Hospital A. The prognostic role of AFI may be modulated by a combination of sex, disease stage and comorbidities, warranting more targeted investigation. One possible explanation is the known sex‐specific differences in visceral fat distribution and its impact on cardiovascular mortality. Kammerlander et al. showed that the VAT/SAT ratio had a stronger association with becoming metabolically unhealthy in women than BMI alone [31]. They showed that quantification of VAT above the S1 vertebral level scans allows more accurate assessment of obesity‐associated cardiometabolic and cardiovascular risk, especially in women. In this context, we observed a possible reversal in male patients with metastatic NSCLC at Hospital B, where an above‐median AFI appeared to be protective. This could potentially be explained by more intensive monitoring and management of cardiovascular risk factors in male patients following cancer diagnosis.

Although not in the focus of the study, no consistent effects of body composition variables on outcome were observed in SCLC patients. The outcome for SCLC patients is generally worse than for NSCLC patients, with relative 5‐year survival rates of only 8.2% for SCLC in the period 2017–2019. This is in line with our data, where the estimated 5‐year survival rate was 13.6% (Hospital A)/10.0% (Hospital B) for SCLC patients as compared to 29.0%/18.6% for NSCLC patients. Metabolic changes or effects of body composition may play a less important role in these patients compared to those with NSCLC, as these effects may not manifest within the typically short survival time. Future studies could benefit from a more detailed analysis of whether meaningful conclusions can be drawn by comparing patients with nonmetastatic tumour stages to those with already advanced disease stages.

Multivariate Cox regression confirmed that higher SI was associated with improved survival, higher MFI was associated with poorer outcomes and the AFI showed no significant association with outcome. This highlights that, in particular, muscle composition and muscle volume in relation to bone volume are significant for prognostic assessments in lung cancer patients. As some studies have shown, this could provide a data basis for decisions regarding potential interventions, such as targeted muscle building or nutritional aspects [32].

Our machine learning‐based survival model, trained on data from Hospital A, showed robust predictive performance in both internal and external validation cohorts. The inclusion of one body composition marker (SI) improved risk stratification beyond conventional clinical parameters, reinforcing their value for prognostic modelling. ECOG performance status and M‐status were the most important factors. Notably, SI outperformed L3‐based SMI in our multivariate models, suggesting it may serve as a more sensitive surrogate for overall health status and physiologic reserve in lung cancer patients, particularly in those with low ECOG scores (ECOG 0–2), where SI may provide a more nuanced understanding of prognosis beyond standard assessments such as ECOG and BMI. Follow‐up studies should investigate possible multicollinearity effects among predictors and the independent contribution of each feature to the prognostic model.

It is important to note that our analysis was limited to CT scans acquired at the time of initial staging, prior to the initiation of cancer‐specific therapy. This approach does not account for the wide range of treatment options that may affect body composition over time. Previous studies suggest that longitudinal monitoring of muscle and fat compartments can provide valuable insights into treatment effects and response to supportive interventions [33, 34].

One challenge in interpreting clinical results on body composition and its impact on lung cancer survival is the common use of metformin for type II diabetes, prevalent in overweight and obese patients. Metformin, studied for its anticancer and immunomodulatory effects, shows variable potency in preclinical and clinical studies [35] [S7].

A notable limitation of our study was the significant disparity in overall survival distributions between the two participating centres. Patients from Hospital B exhibited generally shorter survival times, likely attributable to their higher median ECOG performance status, indicating greater functional impairment at baseline. This systematic difference may have weakened the statistical power to detect subtle prognostic effects in this cohort, potentially masking smaller but clinically relevant associations between body composition markers and survival outcomes. This highlights the importance of accounting for performance status as a confounding variable when validating imaging biomarkers across heterogeneous patient populations.

Another limitation was the limited availability of additional clinical data: While we incorporated BMI, ECOG, metastatic status and L3‐based single‐slice measurements (SMI, SATI, VATI and IMATI), these parameters were only readily available for a limited patient subset, constraining more extensive analysis. Consequently, we refrained from including additional potentially relevant variables such as systemic inflammatory markers (e.g., albumin, CRP and LDH), comorbidities and treatment‐related variables. Future studies should also integrate tumour‐specific variables such as stage, molecular profile and histology to better contextualize body composition effects.

Beyond standard diagnostic applications, CT datasets contain valuable information on body composition that has yet to be fully utilized. This study aims to establish the foundational work for leveraging this untapped data. In the context of nationally planned structured lung cancer screening, increasing amounts of data will be generated that require standardized evaluation. Previous studies have demonstrated that BCA can also be applied to low‐dose chest CT scans, allowing the extraction of predictive markers associated with mortality [36]. This would provide an additional benefit for participants in lung cancer screening programmes, offering early tumour detection and insights into their overall health and prognosis through body composition assessment.

BCA could enhance clinical practice through multiple applications: Patients with unfavourable body composition profiles could benefit from early interventions, such as targeted nutritional or exercise programmes. Additionally, incorporating body composition parameters into pharmacokinetic studies may enable more individualized dosing strategies, especially for cytotoxic agents, where lean and fat mass impact drug metabolism. Lastly, BCA could enable risk‐stratified trial design and the monitoring of treatment‐related changes in body composition over time [37, 38].

Fully‐automated BCA can be easily integrated into existing workflows. With an appropriate server infrastructure in place, the application is cost‐effective compared to other methods, such as DXA scanning. Importantly, fully‐automated BCA is based on CT scans already acquired during the mandatory initial staging of lung cancer and therefore does not involve additional radiation exposure or time‐consuming procedures for the patient.

We believe that image‐based markers will play an increasingly important role in the diagnosis and treatment planning of lung cancer and may also be useful in designing clinical trials to allocate patients to specific study arms, investigating the various interactions of body composition with different therapy regimes. Our upcoming multicentre and European research will not only involve longitudinal BCA observations but also incorporate other image‐based markers like whole‐body organ volumetry.

## 5 Conclusion

Fully automated, volumetric CT‐based BCA was successfully applied and validated to identify novel prognostic biomarkers in lung cancer patients. In particular, the SI showed consistent associations with overall survival and outperformed conventional measures such as L3‐based SMI. Notably, a prognostic model trained on data from Hospital A demonstrated robust performance when externally validated on an independent cohort from Hospital B, underscoring the reproducibility and clinical potential of this approach. These findings support the integration of automated BCA into routine clinical workflows, enabling personalized treatment strategies and laying the groundwork for future studies investigating longitudinal body composition changes and their interaction with therapeutic response and systemic factors. Additionally, BCA could inform decisions regarding potential interventions, such as targeted muscle building, nutritional optimization or cardiometabolic risk management in high‐risk patients.

## Ethics Statement

This study was approved by the Ethics Committees of Hospital A (approval number 21‐10204‐BO) and Hospital B (approval number 2023‐425‐b‐S). Due to the study's retrospective nature, the requirement of written informed consent was waived by the Ethics Committee. All data were fully anonymized before being included in the study.

## Conflicts of Interest

Marcel Kemper received research or travel grants from Amgen, AstraZeneca, Daiichi Sankyo, Janssen‐Cilag, Novartis, Roche Pharma and Takeda Pharma.

Annalen Bleckmann received honoraria or travel grants not related to this manuscript from Bayer, BMS, Takeda, Onkowissen TV, MSD, Boehringer, AstraZeneca, Sanofi, Pfizer, Streamedup, Lilly, Art Tempi, Amgen, RG GmbH, Roche, Novartis, Digimed Verlag, Janssen, DGHO Juniorakademie, Ärztekammer, Pius Hospital Osnabrück, St. Johannes Hospital DO, WTZ, Daiichi, FOMF and Knappschaftskrankenhaus Bochum.

Annalen Bleckmann participated on a Data Safety Monitoring Board or Advisory Board from Bayer, BMS, Takeda, Onkowissen TV, MSD, Boehringer, AstraZeneca, Sanofi, Pfizer, Streamedup, Lilly, Art Tempi, Amgen, RG GmbH, Roche, Novartis, Digimed Verlag, Janssen, Daiichi.

Marcel Opitz received honoraria for Advisory Board not related to this manuscript from Insmed.

Apart from this, the authors declare that they have no known competing financial interests or personal relationships that could have appeared to influence the work reported in this paper.

## Supporting information


**Table S1.** Detailed Kaplan–Meier estimator statistics.
**Table S2.** Detailed Cox regression covariates.
**Figure S1.** Kaplan–Meier survival functions plots when dividing the patient groups by the median of the respective biomarker for SCLC. SCLC: small‐cell lung cancer.
**Figure S2.** Distributions of various measurements, features and indices per sex, centre and cancer subtype.
