# Supplementary material for: Exploration of Fully‐Automated Body Composition Analysis Using Routine CT‐Staging of Lung Cancer Patients for Survival Prognosis
Source: J Cachexia Sarcopenia Muscle. 2025 Aug 6;16(4):e70021. doi: 10.1002/jcsm.70021 (PMC12327357; doi:10.1002/jcsm.70021)
Supplement: Supplementary file 1 — Table S1. Detailed Kaplan–Meier estimator statistics. Table S2. Detailed Cox regression covariates. Figure S1. Kaplan–Meier survival functions plots when dividing the patient groups by the median of the respective biomarker for SCLC. SCLC: small‐cell lung cancer. Figure S2. Distributions of various measurements, features and indices per sex, centre and cancer subtype. [file JCSM-16-e70021-s001.docx]

## Supplement

#### Supplement Table 1 - Detailed Kaplan-Meier estimator statistics

| Subtype | Marker | Sex | M-Status | Hospital A | | | | | Hospital B | | | | |
| --- | --- | --- | --- | --- | --- | --- | --- | --- | --- | --- | --- | --- | --- |
|  |  |  |  | p-  value | Median Survival Time (months) | | Marker median | n | p-  value | Median Survival Time (months) | | Marker median | n |
|  |  |  |  |  | < marker median | ≥ marker median |  |  |  | < marker median | ≥ marker median |  |  |
| NSCLC | SI | all | all | ≤0.001 | 21.4 | 30.7 | 2,18 | 2873 | ≤0.001 | 12.9 | 17.2 | 2,20 | 1198 |
|  |  |  | M0 | ≤0.001 | 30.3 | 46.1 | 2,18 | 1794 | ≤0.001 | 16.7 | 28.8 | 2,18 | 766 |
|  |  |  | M1 | ≤0.001 | 10.8 | 18.2 | 2,19 | 1079 | 0.606 | 10.2 | 10.4 | 2,23 | 432 |
|  |  | m | all | ≤0.001 | 17.1 | 29.1 | 2,26 | 1721 | ≤0.001 | 10.9 | 15.2 | 2,23 | 747 |
|  |  |  | M0 | ≤0.001 | 24.6 | 46.0 | 2,24 | 1079 | ≤0.001 | 13.3 | 28.9 | 2,20 | 485 |
|  |  |  | M1 | ≤0.001 | 8.9 | 16.8 | 2,28 | 642 | 0.037 | 7.0 | 10.4 | 2,29 | 262 |
|  |  | f | all | 0.003 | 27.8 | 36.0 | 2,10 | 1152 | 0.226 | 19.2 | 19.2 | 2,15 | 451 |
|  |  |  | M0 | 0.008 | 37.9 | 53.6 | 2,10 | 715 | 0.018 | 23.0 | 28.6 | 2,14 | 281 |
|  |  |  | M1 | 0.032 | 15.8 | 22.8 | 2,11 | 437 | 0.650 | 13.5 | 14.9 | 2,15 | 170 |
|  | MFI | all | all | ≤0.001 | 31.7 | 21.3 | 2,35 | 2873 | 0.011 | 16.1 | 13.4 | 2,51 | 1198 |
|  |  |  | M0 | ≤0.001 | 49.2 | 29.9 | 2,39 | 1794 | ≤0.001 | 27.1 | 18.7 | 2,60 | 766 |
|  |  |  | M1 | ≤0.001 | 19.3 | 10.3 | 2,29 | 1079 | 0.392 | 11.5 | 9.5 | 2,41 | 432 |
|  |  | m | all | ≤0.001 | 27.4 | 19.2 | 2,44 | 1721 | 0.023 | 14.5 | 11.5 | 2,60 | 747 |
|  |  |  | M0 | ≤0.001 | 43.7 | 28.2 | 2,47 | 1079 | ≤0.001 | 28.8 | 14.5 | 2,67 | 485 |
|  |  |  | M1 | ≤0.001 | 15.8 | 9.0 | 2,37 | 642 | 0.356 | 10.3 | 8.1 | 2,43 | 262 |
|  |  | f | all | 0.028 | 35.4 | 28.5 | 2,22 | 1152 | 0.408 | 19.3 | 19.1 | 2,37 | 451 |
|  |  |  | M0 | 0.009 | 53.6 | 38.5 | 2,24 | 715 | 0.450 | 25.5 | 27.2 | 2,36 | 281 |
|  |  |  | M1 | 0.142 | 21.0 | 15.8 | 2,20 | 437 | 0.676 | 14.9 | 13.1 | 2,38 | 170 |
|  | AFI | all | all | ≤0.001 | 28.9 | 22.7 | 0,59 | 2873 | 0.140 | 17.0 | 13.3 | 0,62 | 1198 |
|  |  |  | M0 | 0.008 | 43.3 | 34.2 | 0,60 | 1794 | 0.097 | 24.5 | 19.9 | 0,63 | 766 |
|  |  |  | M1 | 0.004 | 16.2 | 11.9 | 0,57 | 1079 | 0.139 | 11.7 | 8.8 | 0,59 | 432 |
|  |  | m | all | 0.451 | 21.3 | 24.1 | 0,79 | 1721 | 0.307 | 11.9 | 13.8 | 0,79 | 747 |
|  |  |  | M0 | 0.628 | 31.8 | 34.3 | 0,79 | 1079 | 0.329 | 19.9 | 18.9 | 0,81 | 485 |
|  |  |  | M1 | 0.703 | 11.0 | 12.2 | 0,79 | 642 | 0.042 | 8.1 | 9.6 | 0,77 | 262 |
|  |  | f | all | 0.003 | 36.0 | 28.0 | 0,35 | 1152 | 0.110 | 21.2 | 17.9 | 0,34 | 451 |
|  |  |  | M0 | ≤0.001 | 69.3 | 33.7 | 0,36 | 715 | 0.238 | 25.9 | 25.9 | 0,34 | 281 |
|  |  |  | M1 | 0.263 | 19.0 | 16.4 | 0,34 | 437 | 0.187 | 18.8 | 12.1 | 0,34 | 170 |
| SCLC | SI | all | all | 0.213 | 12.5 | 17.5 | 2,20 | 472 | 0.171 | 10.4 | 12.4 | 2,26 | 166 |
|  |  |  | M0 | 0.306 | 19.3 | 24.6 | 2,20 | 212 | 0.208 | 15.1 | 23.1 | 2,25 | 89 |
|  |  |  | M1 | 0.181 | 10.6 | 12.0 | 2,24 | 260 | 0.268 | 7.8 | 9.5 | 2,28 | 77 |
|  |  | m | all | 0.030 | 11.0 | 17.2 | 2,29 | 275 | 0.071 | 9.8 | 13.1 | 2,38 | 87 |
|  |  |  | M0 | 0.038 | 11.9 | 22.0 | 2,28 | 123 | 0.939 | 19.4 | 19.9 | 2,43 | 50 |
|  |  |  | M1 | 0.255 | 10.6 | 11.8 | 2,29 | 152 | 0.198 | 7.1 | 8.8 | 2,28 | 37 |
|  |  | f | all | 0.386 | 15.6 | 18.5 | 2,09 | 197 | 0.539 | 10.8 | 12.4 | 2,17 | 79 |
|  |  |  | M0 | 0.563 | 21.3 | 32.7 | 2,05 | 89 | 0.041 | 15.1 | 31.6 | 2,10 | 39 |
|  |  |  | M1 | 0.153 | 10.6 | 14.2 | 2,13 | 108 | 0.797 | 10.8 | 10.5 | 2,29 | 40 |
|  | MFI | all | all | 0.887 | 17.5 | 12.5 | 2,35 | 472 | 0.099 | 13.0 | 10.3 | 2,52 | 166 |
|  |  |  | M0 | 0.923 | 20.7 | 21.1 | 2,40 | 212 | 0.461 | 19.9 | 15.1 | 2,54 | 89 |
|  |  |  | M1 | 0.251 | 12.4 | 10.0 | 2,33 | 260 | 0.090 | 10.5 | 6.9 | 2,45 | 77 |
|  |  | m | all | 0.781 | 14.1 | 11.1 | 2,38 | 275 | 0.550 | 13.0 | 10.4 | 2,53 | 87 |
|  |  |  | M0 | 0.402 | 20.6 | 17.8 | 2,43 | 123 | 0.671 | 19.4 | 20.1 | 2,52 | 50 |
|  |  |  | M1 | 0.839 | 11.7 | 10.6 | 2,35 | 152 | 0.231 | 8.8 | 7.1 | 2,53 | 37 |
|  |  | f | all | 0.923 | 18.5 | 15.2 | 2,32 | 197 | 0.047 | 13.0 | 7.8 | 2,45 | 79 |
|  |  |  | M0 | 0.789 | 27.9 | 32.7 | 2,37 | 89 | 0.115 | 26.3 | 9.8 | 2,65 | 39 |
|  |  |  | M1 | 0.124 | 14.9 | 8.6 | 2,32 | 108 | 0.217 | 10.5 | 5.8 | 2,38 | 40 |
|  | AFI | all | all | 0.055 | 18.1 | 11.9 | 0,55 | 472 | 0.876 | 12.3 | 11.5 | 0,51 | 166 |
|  |  |  | M0 | 0.008 | 32.2 | 15.9 | 0,55 | 212 | 0.824 | 14.9 | 20.1 | 0,54 | 89 |
|  |  |  | M1 | 0.456 | 10.8 | 11.0 | 0,54 | 260 | 0.109 | 10.5 | 7.1 | 0,50 | 77 |
|  |  | m | all | 0.997 | 15.9 | 11.9 | 0,77 | 275 | 0.192 | 12.3 | 10.6 | 0,71 | 87 |
|  |  |  | M0 | 0.893 | 20.6 | 13.7 | 0,77 | 123 | 0.126 | 24.4 | 14.8 | 0,70 | 50 |
|  |  |  | M1 | 0.971 | 10.7 | 11.9 | 0,77 | 152 | 0.976 | 8.5 | 9.5 | 0,75 | 37 |
|  |  | f | all | 0.426 | 19.3 | 14.9 | 0,35 | 197 | 0.034 | 8.8 | 13.9 | 0,32 | 79 |
|  |  |  | M0 | 0.806 | 28.0 | 27.1 | 0,38 | 89 | 0.072 | 9.8 | 26.5 | 0,35 | 39 |
|  |  |  | M1 | 0.068 | 17.2 | 8.6 | 0,33 | 108 | 0.375 | 7.8 | 10.5 | 0,32 | 40 |

####

####

#### Supplement Table 2 - Detailed Cox Regression Covariates

|  | Hospital | marker | covariate | HR (95% CI) | p-value |
| --- | --- | --- | --- | --- | --- |
| NSCLC | A | Sarcopenia (SI) | marker | 0.53 (0.46-0.62) | ≤0.001 |
|  |  |  | age | 1.01 (1.00-1.01) | 0.070 |
|  |  |  | sex | 0.67 (0.60-0.75) | ≤0.001 |
|  |  |  | M Status | 2.44 (2.20-2.71) | ≤0.001 |
|  |  | Myosteatotic Fat (MFI) | marker | 1.31 (1.22-1.40) | ≤0.001 |
|  |  |  | age | 1.01 (1.01-1.02) | ≤0.001 |
|  |  |  | sex | 0.78 (0.70-0.87) | ≤0.001 |
|  |  |  | M Status | 2.43 (2.18-2.70) | ≤0.001 |
|  |  | Abdominal Fat (AFI) | marker | 1.01 (0.81-1.26) | 0.929 |
|  |  |  | age | 1.02 (1.01-1.02) | ≤0.001 |
|  |  |  | sex | 0.77 (0.66-0.89) | ≤0.001 |
|  |  |  | M Status | 2.37 (2.13-2.64) | ≤0.001 |
|  | B | Sarcopenia (SI) | marker | 0.59 (0.48-0.72) | ≤0.001 |
|  |  |  | age | 1.00 (0.99-1.01) | 0.733 |
|  |  |  | sex | 0.71 (0.61-0.83) | ≤0.001 |
|  |  |  | M Status | 1.81 (1.57-2.09) | ≤0.001 |
|  |  | Myosteatotic Fat (MFI) | marker | 1.12 (1.04-1.21) | 0.002 |
|  |  |  | age | 1.01 (1.00-1.02) | 0.008 |
|  |  |  | sex | 0.78 (0.67-0.90) | ≤0.001 |
|  |  |  | M Status | 1.81 (1.57-2.09) | ≤0.001 |
|  |  | Abdominal Fat (AFI) | marker | 0.90 (0.70-1.16) | 0.419 |
|  |  |  | age | 1.01 (1.00-1.02) | 0.001 |
|  |  |  | sex | 0.75 (0.62-0.90) | 0.003 |
|  |  |  | M Status | 1.78 (1.54-2.05) | ≤0.001 |
| SCLC | A | Sarcopenia (SI) | marker | 0.69 (0.52-0.92) | 0.011 |
|  |  |  | age | 1.02 (1.00-1.03) | 0.008 |
|  |  |  | sex | 0.81 (0.64-1.03) | 0.086 |
|  |  |  | M Status | 2.04 (1.61-2.59) | ≤0.001 |
|  |  | Myosteatotic Fat (MFI) | marker | 1.28 (1.08-1.51) | 0.004 |
|  |  |  | age | 1.02 (1.01-1.03) | 0.006 |
|  |  |  | sex | 0.89 (0.71-1.12) | 0.323 |
|  |  |  | M Status | 2.03 (1.61-2.58) | ≤0.001 |
|  |  | Abdominal Fat (AFI) | marker | 0.87 (0.55-1.38) | 0.560 |
|  |  |  | age | 1.02 (1.01-1.04) | ≤0.001 |
|  |  |  | sex | 0.83 (0.61-1.12) | 0.216 |
|  |  |  | M Status | 1.93 (1.52-2.44) | ≤0.001 |
|  | B | Sarcopenia (SI) | marker | 0.73 (0.44-1.22) | 0.228 |
|  |  |  | age | 1.02 (1.00-1.04) | 0.073 |
|  |  |  | sex | 0.93 (0.64-1.35) | 0.703 |
|  |  |  | M Status | 2.25 (1.56-3.24) | ≤0.001 |
|  |  | Myosteatotic Fat (MFI) | marker | 1.11 (0.93-1.34) | 0.251 |
|  |  |  | age | 1.03 (1.00-1.05) | 0.015 |
|  |  |  | sex | 0.98 (0.69-1.40) | 0.910 |
|  |  |  | M Status | 2.28 (1.58-3.30) | ≤0.001 |
|  |  | Abdominal Fat (AFI) | marker | 0.91 (0.37-2.23) | 0.841 |
|  |  |  | age | 1.03 (1.01-1.05) | 0.008 |
|  |  |  | sex | 0.96 (0.60-1.54) | 0.861 |
|  |  |  | M Status | 2.22 (1.54-3.20) | ≤0.001 |


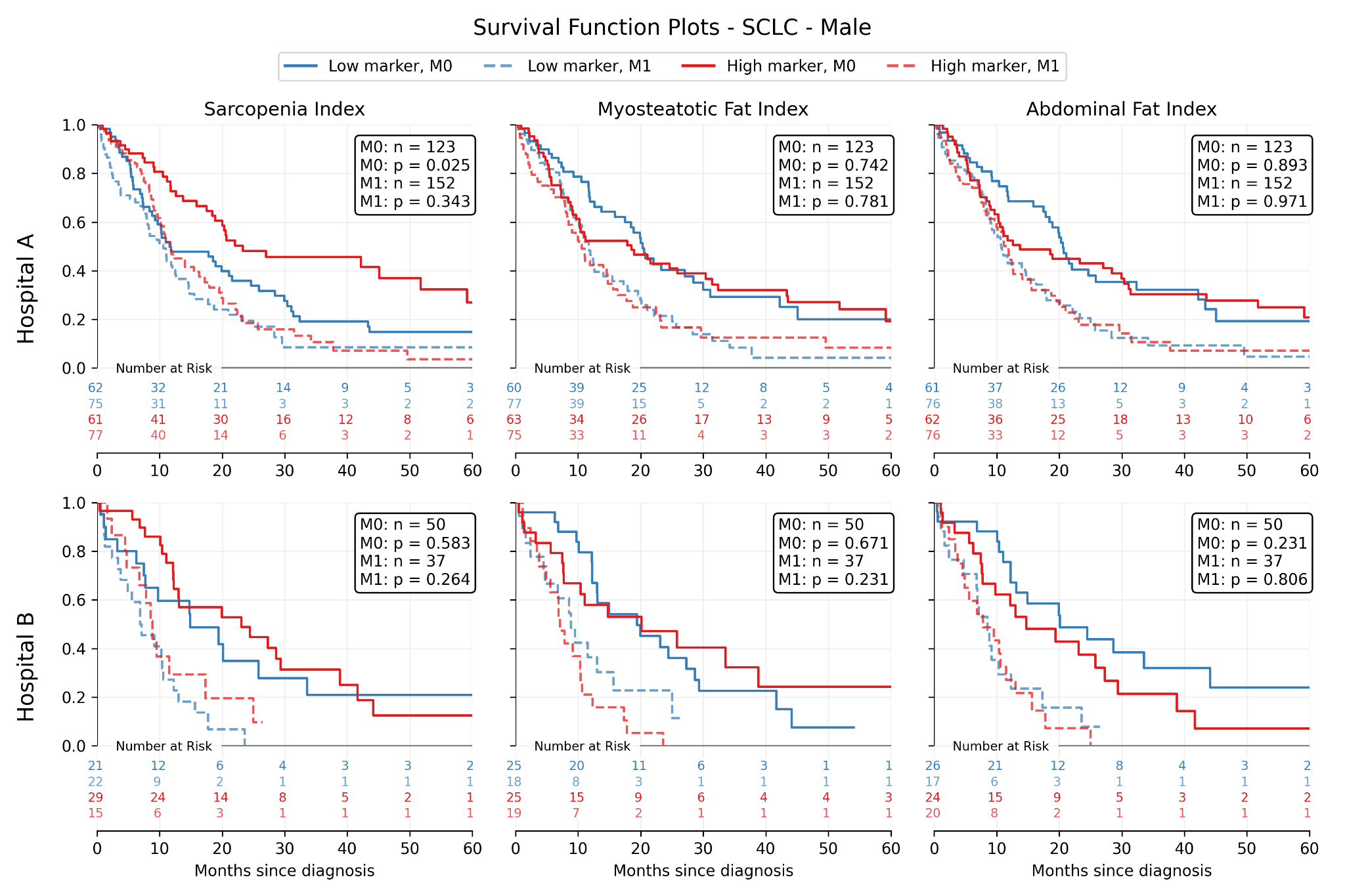


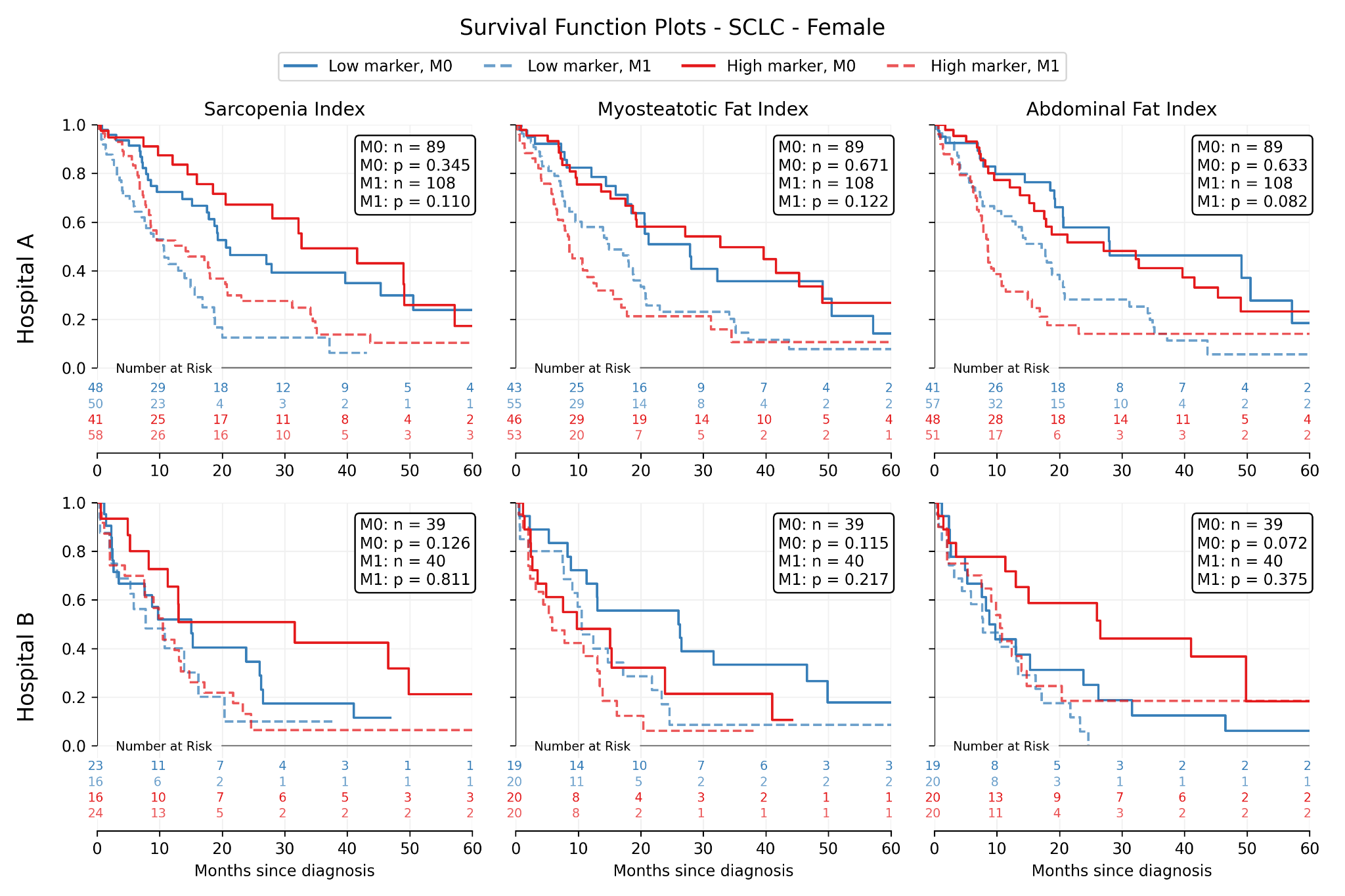


**Supplement Fig. 1** Kaplan-Meier survival functions plots when dividing the patient groups by the median of the respective biomarker for SCLC. SCLC: Small-Cell Lung Cancer


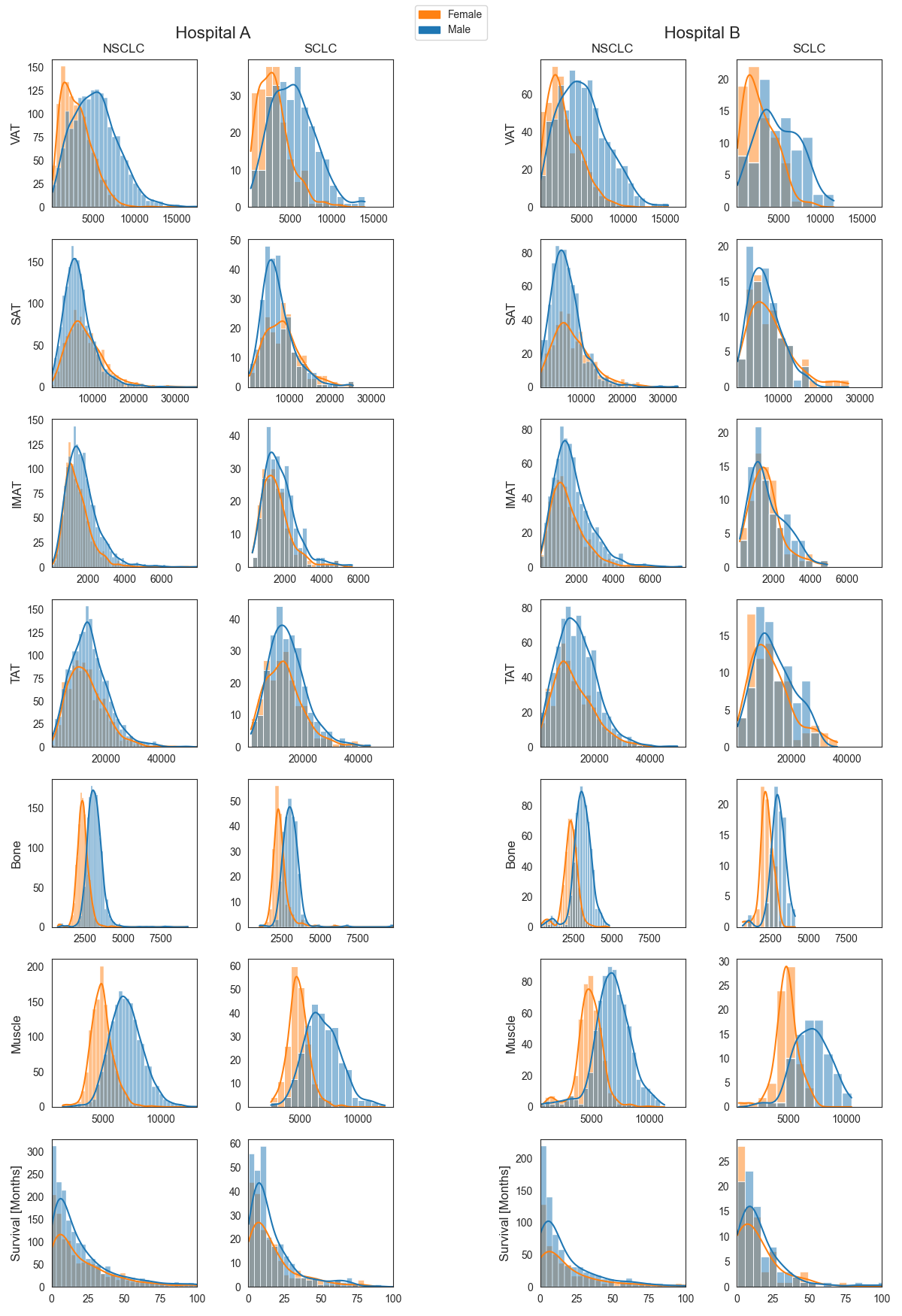

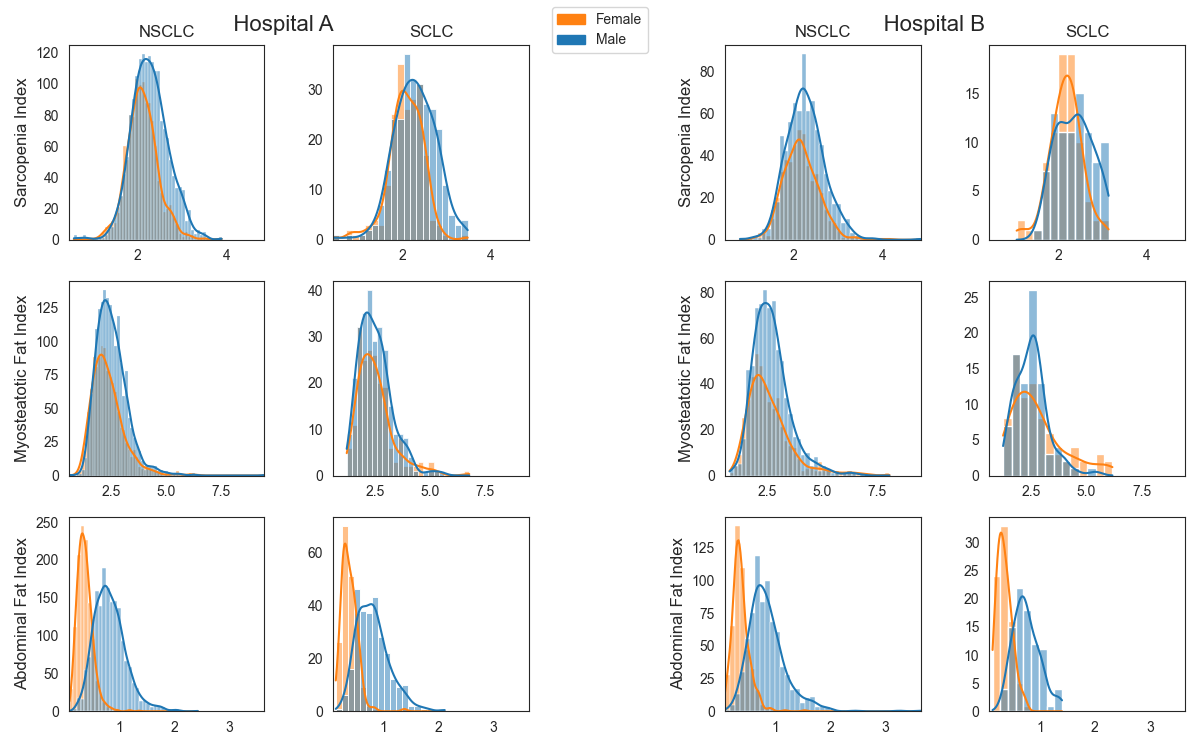


**Supplement Fig. 2** Distributions of various measurements, features, and indices per sex, center, and cancer subtype.

####

#### Supplement References

S1 - [Ibrahim MM. Subcutaneous and visceral adipose tissue: structural and functional differences. *Obes Rev*. 2010;11(1):11-18. doi:10.1111/j.1467-789X.2009.00623.x](https://www.zotero.org/google-docs/?a4fbdV)

S2 - [Chen JL, Walton KL, Qian H, et al. Differential Effects of IL6 and Activin A in the Development of Cancer-Associated Cachexia. *Cancer Res*. 2016;76(18):5372-5382. doi:10.1158/0008-5472.CAN-15-3152](https://www.zotero.org/google-docs/?a4fbdV)

S3 -  [Liu W, Wang H, Bai F, et al. IL-6 promotes metastasis of non-small-cell lung cancer by up-regulating TIM-4 via NF-κB. *Cell Prolif*. 2020;53(3):e12776. doi:10.1111/cpr.12776](https://www.zotero.org/google-docs/?a4fbdV)

S4 - [Batista ML, Neves RX, Peres SB, et al. Heterogeneous time-dependent response of adipose tissue during the development of cancer cachexia. *J Endocrinol*. 2012;215(3):363-373. doi:10.1530/JOE-12-0307](https://www.zotero.org/google-docs/?a4fbdV)

S5 - [Baazim H, Antonio-Herrera L, Bergthaler A. The interplay of immunology and cachexia in infection and cancer. *Nat Rev Immunol*. 2022;22(5):309-321. doi:10.1038/s41577-021-00624-w](https://www.zotero.org/google-docs/?a4fbdV)

S6 - [Ardesch FH, Ruiter R, Mulder M, Lahousse L, Stricker BHC, Kiefte-de Jong JC. The Obesity Paradox in Lung Cancer: Associations With Body Size Versus Body Shape. *Front Oncol*. 2020;10:591110. doi:10.3389/fonc.2020.591110](https://www.zotero.org/google-docs/?a4fbdV)

S7 - [Eikawa S, Nishida M, Mizukami S, Yamazaki C, Nakayama E, Udono H. Immune-mediated antitumor effect by type 2 diabetes drug, metformin. *Proc Natl Acad Sci*. 2015;112(6):1809-1814. doi:10.1073/pnas.1417636112](https://www.zotero.org/google-docs/?a4fbdV)

S8 - [Tsukamoto M, Imai K, Ishimoto T, et al. PD ‐L1 expression enhancement by infiltrating macrophage‐derived tumor necrosis factor‐α leads to poor pancreatic cancer prognosis. *Cancer Sci*. 2019;110(1):310-320. doi:10.1111/cas.13874](https://www.zotero.org/google-docs/?a4fbdV)

S9 - [Zhang W, Tang J, Tang H, et al. Different computed tomography parameters for defining myosteatosis in patients with advanced non-small cell lung cancer. *Clin Nutr*. 2023;42(12):2414-2421. doi:10.1016/j.clnu.2023.10.006](https://www.zotero.org/google-docs/?a4fbdV)

S10 -  [Lee CM, Kang J. Prognostic impact of myosteatosis in patients with colorectal cancer: a systematic review and meta‐analysis. *J Cachexia Sarcopenia Muscle*. 2020;11(5):1270-1282. doi:10.1002/jcsm.12575](https://www.zotero.org/google-docs/?a4fbdV)
